# Comprehensive analysis of m^7^G modification patterns based on potential m^7^G regulators and tumor microenvironment infiltration characterization in lung adenocarcinoma

**DOI:** 10.3389/fgene.2022.996950

**Published:** 2022-09-29

**Authors:** Shouzheng Ma, Jun Zhu, Mengmeng Wang, Jianfei Zhu, Wenchen Wang, Yanlu Xiong, Runmin Jiang, Lei Liu, Tao Jiang

**Affiliations:** ^1^ Department of Thoracic Surgery, Tangdu Hospital, Fourth Military Medical University, Xi’an, China; ^2^ Department of General Surgery, The Southern Theater Air Force Hospital, Guangzhou, China; ^3^ Department of Drug and Equipment, Lintong Rehabilitation and Convalescent Centre, Xi’an, China; ^4^ Department of Gastroenterology, Tangdu Hospital, Fourth Military Medical University, Xi’an, China; ^5^ Department of Gastroenterology, Daping Hospital, Army Medical University, Chongqing, China

**Keywords:** lung adenocarcinoma, tumor microenvironment, mutation burden, immunotherapy, N7-methylguanosine (m^7^G)

## Abstract

**Background:** The non-negligible role of epigenetic modifications in cancer development and tumor microenvironment (TME) has been demonstrated in recent studies. Nonetheless, the potential regulatory role of N7-methylguanosine (m^7^G) modification in shaping and impacting the TME remains unclear.

**Methods:** A comprehensive analysis was performed to explore the m^7^G modification patterns based on 24 potential m^7^G regulators in 817 lung adenocarcinoma (LUAD) patients, and the TME landscape in distinct m^7^G modification patterns were evaluated. The m^7^G score was established based on principal component analysis (PCA) to quantify m^7^G modification patterns and evaluate the TME cell infiltrating characteristics of individual tumors. Further, correlation analyses of m7Gscore with response to chemotherapy and immunotherapy were performed.

**Results:** We identified three distinct m^7^G modification patterns with the biological pathway enrichment and TME cell infiltrating characteristics corresponded to immune-desert, immune-inflamed and immune-excluded phenotype, respectively. We further demonstrated the m^7^Gscore could predict the TME infiltrating characteristics, tumor mutation burden (TMB), response to immunotherapy and chemotherapy, as well as prognosis of individual tumors. High m^7^Gscore was associated with increased component of immune cell infiltration, low TMB and survival advantage, while low m^7^Gscore was linked to decreased immune cell infiltration and increased TMB. Additionally, patients with lower m^7^Gscore demonstrated significant therapeutic advantages.

**Conclusion:** This study demonstrated the regulatory mechanisms of m^7^G modification on TME formation and regulation of lung adenocarcinoma. Identification of individual tumor m^7^G modification patterns will contribute to the understanding of TME characterization and guiding more effective immunotherapy strategies.

## Introduction

Lung cancer is currently the second most frequently diagnosed cancer, and the leading cause of cancer death in the world, accounting for 11.4% of all new cancer diagnoses and causing 18.7% of cancer-related deaths (Sung et al., 2021). Lung adenocarcinoma (LUAD) is the most common subtype, accounting for approximately 40% of all lung cancers. Current clinical treatments of LUAD include surgery, chemotherapy, radiotherapy, immunotherapy and molecularly targeted agents. Unfortunately, the poor prognosis of patients highlights the urgent need for the development of novel and more specific therapeutic targets for the treatment of LUAD. Accumulated evidence suggests that aberrant epigenetic modifications, especially RNA methylation, play critical roles in cancer development and progression (Berdasco and Esteller, 2010).

N7-methylguanosine (m^7^G) is one of the most prevalent modifications occurring in transfer RNA (tRNA) (Gauss et al., 1979), ribosomal RNA (rRNA) (Motorin and Helm, 2011) and messenger RNA (mRNA) 5′cap (Capping, 1976), that plays an essential role in regulating RNA processing, exporting, metabolism and function. Meanwhile, as a universally conserved modified nucleosides, m^7^G was found widely among eubacteria, eukaryotes (Juhling et al., 2009), and a few archaea (Edmonds et al., 1991). Notably, recent researches have begun to demonstrate the existence of m^7^G modification within internal mRNAs in higher eukaryotes (Chu et al., 2018; Malbec et al., 2019), and identified the distribution features of the internal mRNA m^7^G using both methylated RNA immunoprecipitation sequencing and chemical modification-assisted BS-seq methods (Zhang et al., 2019). Accumulative evidences have unraveled part of the regulatory mechanisms of m^7^G modification within mRNA, for example, METTL1-WDR4 complex was demonstrated to act as the m^7^G methyltransferases for mRNAs and mediate their formation (Malbec et al., 2019). Thus, m^7^G has become a novel biological marker with critical regulatory roles with the rapid advancement of sequencing technology. To further investigate the m^7^G modification patterns in LUAD and elucidate their impact on tumor progression, we retrieved 24 potential m^7^G modification-related regulators by considering the previous research (Tomikawa, 2018) and exploring the Molecular Signatures Database (www.broadinstitute.org/gsea/msigdb/annotate.jsp).

Tumor progression depends not just on the genetic and epigenetic heterogeneity of tumor cells, but also on the tumor microenvironment (TME), a complex environment containing tumor cells, interstitial cells [e.g., fibroblasts, endothelial cells, tumor-associated macrophages (TAMs)], distant recruited cells [e.g., infiltrating immune cells and bone marrow-derived cells (BMDCs)], and non-cellular elements (e.g., extracellular matrix, cytokines, chemokines and new blood vessels) (Witz and Levy-Nissenbaum, 2006). Complex interactions between tumor and TME are critically involved in multiple malignant biological behaviors such as stimulating cells proliferation and angiogenesis, suppressing apoptosis, as well as inducing immune tolerance (Mantovani et al., 2008). Growing evidence reveals the pivotal role of TME in tumorigenesis, tumor progression and immune evasion (Quail and Joyce, 2013). In particular, TME significantly correlate with response to immune checkpoint blockade (ICB) therapy, and the evaluation of TME cell infiltrating characterization is crucial for the development of novel immunotherapeutic strategies (Ali et al., 2016). Thus, comprehensive analyses of the TME landscape facilitate the identification of distinct tumor immunophenotypes, and contribute to developing biomarkers of the response to immunotherapy and discovering novel targets for immunotherapy.

Compelling evidence has revealed the pivotal role of RNA methylation in shaping and impacting the TME (especially immune cells infiltrating) (Cao and Yan, 2020; Zhang et al., 2020). The m6A RNA methylation has been reported mediating the biological behavior of tumor cells and tumor-infiltrating immune cells by regulating RNA splicing, translation, initiation degradation and nuclear export (Pinello et al., 2018; Han et al., 2019; Wang et al., 2019). And m1A methylation modification has been confirmed to participate in the regulation of TME complexity and diversity based on immune cell infiltration (Gao et al., 2021; Liu et al., 2021). Chen et al. (2022) reported that METTL1-mediated m^7^G modification altered the immune characterization and dynamic interplay between tumor cells and surrounding stromal compartment. Galloway et al. (2021) reported that m^7^G cap methyltransferase RNMT increased translational capacity during T cell activation by coordinating mRNA processing.

However, due to methodological limitations, these studies have necessarily focused on one or several m^7^G regulators and cell types, while the antitumor effect of RNA modification is a highly coordinated process that regulated by numerous tumor suppressor factors. Therefore, a comprehensive analysis of the correlation between TME cell infiltration characterizations and multiple m^7^G regulators will further elucidated the mechanisms of m^7^G modification regulating the TME characterization and provide novel support for more effective immunotherapy.

In this study, we extracted and integrated the genomic data of 817 LUAD samples from the public databases to comprehensively analyze the m^7^G modification patterns, as well as explored the TME cell infiltrating characteristics under different patterns. We identified three m^7^G modification patterns which corresponded to immune-desert, immune-inflamed and immune-excluded phenotype, respectively, revealing that m^7^G modification played an indispensable role in shaping the TME characterization. Furthermore, a set of scoring system was established to quantify the individual tumor m^7^G modification patterns in LUAD patients. Improving the m^7^G modification patterns by targeting m^7^G regulators or m^7^G-related genes may alter TME cell-infiltrating characteristics, that may contribute to the development of novel immunotherapy target or optimization of combination therapy strategies.

## Materials and methods

### Data extraction and preprocessing

**FIGURE 1:**
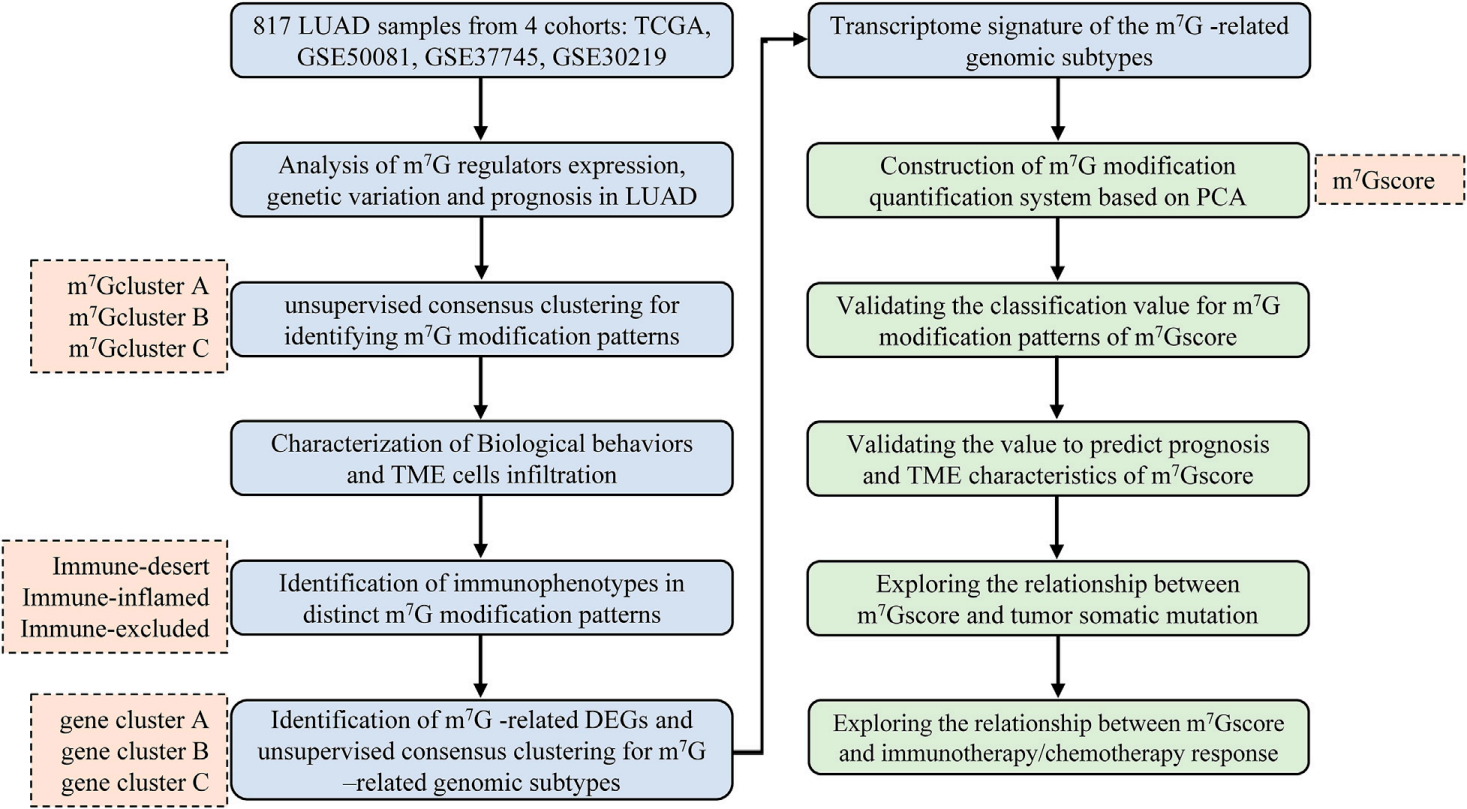
Overview of the study design and analytical flow. LUAD, lung adenocarcinoma; m^7^G, N7-methylguanosine; TME, tumor microenvironment; DEG, differentially expressed gene; PCA, principal component analysis.

The flowchart of this study was shown in Figure 1. Gene expression profiles and matching clinical annotation were retrieved from The Cancer Genome Atlas (TCGA, https://www.cancer.gov/) and Gene Expression Omnibus (GEO, https://www.ncbi.nlm.nih.gov/geo/) database. Patients with incomplete survival information were excluded from further analyses. Finally, 817 LUAD patients were included from four datasets (GSE50081, GSE37745, GSE30219, and TCGA-LUAD) for further analyses in this study. For consistency, Fragments Per Kilobase of transcript per Million reads sequenced (FPKM) values (TCGA-RNA sequencing data) were converted into transcripts per kilobase million (TPM) values. Batch effects in this cohort were removed using the ComBat algorithm (sva R package). The detailed information for each dataset was summarized in Supplementary Table S1. The somatic mutation profiles and Copy Number Variation (CNV) data of LUAD samples were obtained from TCGA database. These data were analyzed using the R (version 4.1.1) and R Bioconductor packages.

### Identification and unsupervised clustering for 7-methylguanosine regulators

Twenty four regulators related to m^7^G modification were identified from the published literature (Tomikawa, 2018) and three Gene Set (M26066, M26714, M18244) from Gene Set Enrichment Analysis (GSEA, http://www.gsea-msigdb.org/gsea/index.jsp), including METTL1, WDR4, NSUN2, DCP2, DCPS, NUDT3, NUDT4, NUDT10, NUDT11, NUDT16, AGO2, CYFIP1, EIF3D, EIF4E, EIF4E2, EIF4E3, EIF4G3, GEMIN5, LARP1, NCBP1, NCBP2, IFIT5, LSM1, and SNUPN. To identify distinct m^7^G modification patterns and categorize patients into subgroups, we performed unsupervised consensus clustering analysis (K-Means algorithm, Euclidean distance measure) using “ConsensusClusterPlus” R package (Hartigan and Wong, 1979; Wilkerson and Hayes, 2010), and conduct 1,000 repetitions to ensure the stability of classification.

### Biological pathway enrichment analysis

To explore the difference in biological pathway between distinct m^7^G modification patterns, we used “GSVA” R package to conduct Gene set variation analysis (GSVA), which is a non-parametric, unsupervised method for estimating variation of gene set enrichment through the samples of an expression dataset (Hänzelmann et al., 2013). The gene sets of “c2. cp.kegg.v7.5.1,” “c5. go.bp.v7.5.1,” “c5. go.cc.v7.5.1,” and “c5. go.mf.v7.5.1” were downloaded from GSEA database for GSVA analysis, and adjusted *p* value less than 0.05 was considered statistically significant.

### Comprehensive analysis of the tumor microenvironment characterization

The Estimation of Stromal and Immune cells in Malignant Tumor tissues using Expression data (ESTIMATE) algorithm (Yoshihara et al., 2013) was performed to infer the fraction of stromal (defined as stromal score) and immune cells (defined as immune score) in each LUAD samples using “ESTIMATE” R package (v1.1.0, https://bioinformatics.mdanderson.org/estimate/rpackage.html). The ESTIMATE score is the sum of the immune score and the stromal score, and represents the comprehensive proportion of both components in the TME. Tumor purity was defined as the percentage of malignant cells in a solid tumor sample. The single-sample gene-set enrichment analysis (ssGSEA) algorithm (Barbie et al., 2009) was performed to quantify the immune cell infiltration using “GSVA” R package. The immune cell population were determined with reference to the study of Zhang (Zhang et al., 2020). Specific marker gene sets for each immune cell type (Supplementary Table S2) were derived from the published literatures (Barbie et al., 2009; Charoentong et al., 2017), which contained both innate immune cells (eg, eosinophils, neutrophils, macrophages) and adaptive immune cells (e.g., CD4^+^ T cell, CD8^+^ T cell, regulatory T cell). The gene set of immune-checkpoints was referred to Mariathasan et al. (2018).

### N7-methylguanosine-related genes identification and N7-methylguanosine gene signature construction

Differentially expressed genes (DEGs) among distinct m^7^G modification patterns were determined by empirical Bayesian method using the “limma” R package (Smyth, 2004), and the selection criteria was set as adjusted *p* value <0.001. The intersections of distinct DEGs were defined as m^7^G-related DEGs. Based on m^7^G-related DEGs, all patients were classified into several subgroups for further analysis by performing unsupervised consensus clustering analysis (K-Means algorithm, Euclidean distance measure). This procedure was repeated 1,000 times to ensure the stability of classification. The “clusterProfiler” R package was used to perform GO enrichment analysis for the m^7^G-related DEGs, and significant enrichment pathways (adjust *p* value <0.05 and Q value <0.05) were displayed in the barplot.

Furthermore, to quantify the m^7^G modification patterns of individual tumors, a m^7^G gene signature (named as m^7^Gscore) was conducted according to the following steps. Firstly, univariate Cox regression analysis was performed to identified significant (*p* < 0.001) prognosis m^7^G-related DEGs. Following principal component analysis (PCA), both principal components 1 (PC1) and principal components 2 (PC2) were extracted to act as the gene signature score. Finally, we applied a method similar to gene expression grade index (GGI) (Sotiriou et al., 2006; Zhang et al., 2020) to define the m^7^Gscore of each patient: $m^{7}Gscore=\sum \left({PC1}_{i}+{PC2}_{i}\right)$ , where i is the expression of m^7^G-related DEGs.

### Prediction of immunotherapy and chemotherapy response

We investigated the predictive capacity of m^7^Gscore in responding immunotherapy and four common first-line chemotherapy drugs (cisplatin, paclitaxel, docetaxel, gemcitabine) (Ettinger et al., 2021) for LUAD. The clinical response to immunotherapy was inferred by the Tumor Immune Dysfunction and Exclusion (TIDE, http://tide.dfci.harvard.edu/), an algorithm to simulate two primary mechanisms of tumor immune evasion: the induction of T cell dysfunction in tumors with high cytotoxic T lymphocytes (CTLs) infiltration and the prevention of T cell infiltration in tumors with low CTL (Jiang et al., 2018). Generally, a lower T cell dysfunction signature score predicts a better response to immunotherapy. The 50% inhibiting concentration (IC50) values of the four chemotherapy drugs were predicted using the pRRophetic algorithm (Geeleher et al., 2014) and the value was normally transformed.

### Statistical analysis

The Student’s *t* test was used to compare the differences between two groups, and one-way ANOVA and Kruskal-Wallis tests were used to compare the differences among multiple groups. Spearman correlation coefficient was used for correlation analysis. Survival curves were constructed using the Kaplan-Meier method, and the log-rank test was used to identify the significance of differences. Univariate Cox regression analysis was performed to estimate the hazard ratios (HR) and 95% confidence intervals (CI). Multivariate Cox regression analysis was employed to identify independent prognostic factors, and only patients with complete clinical information were included in final multivariate analysis. The waterfall plots of a mutational landscape in TCGA-LUAD cohort were generated using “maftools” R package (Mayakonda et al., 2018). The copy number variation (CNV) landscape of m^7^G regulators in 23 pairs of chromosomes was visualized using “RCircos” R package (Zhang et al., 2013). All statistical analyses were performed using R software (version 4.1.1), and a *p* value < 0.05 was considered statistically significant.

## Results

### Analysis of genetic variation, expression and prognostic value of m^7^G regulators in lung adenocarcinoma

**FIGURE 2:**
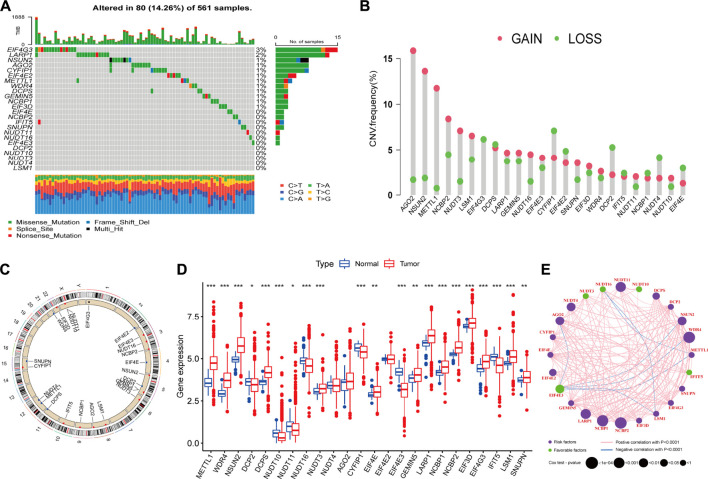
Landscape of genetic and expression variation of m^7^G regulators in lung adenocarcinoma (LUAD). **(A)** The mutation frequency of 24 m^7^G regulators in 561 patients with LUAD from TCGA-LUAD cohort, and each column represents individual patients. The top barplot depicts tumor mutation burden and mutation frequency in each regulator is given on the right. The right barplot depicts the proportion of each variant type. The stacked barplot below depicts fraction of conversions in each sample. **(B)** The copy number variation (CNV) alteration frequency of m^7^G regulators in TCGA-LUAD cohort. The height of each column represents the alteration frequency (Red dot: the amplification frequency; green dot: the deletion frequency). **(C)** The location of CNV alteration of m^7^G regulators on 23 chromosomes using TCGA-LUAD cohort. **(D)** The expression of m^7^G regulators between normal tissues and LUAD tissues (Red: tumor; blue: normal). The horizontal line indicates the median, the lower and upper boundaries of the boxes the interquartile range, and the dots the outliers. Asterisks indicate statistical significance, *p < 0.05, **p < 0.01, ***p < 0.001. **(E)** The interaction between m^7^G regulators in LUAD. Each regulator is represented as a circle, where the size of the circle represents the effect of each regulator on the prognosis, and *p* values were calculated by Log-rank test (*p < 0.05, **p < 0.01, ***p < 0.001).

Genetic alteration is a critical factor influencing gene expression and function, we firstly explored the incidence of somatic mutations and copy number variations (CNV) of 24 m^7^G regulators in LUAD. As shown in Figure 2A, 80 of 561, (14.26%) samples experienced mutations of m^7^G regulators. Among them, the mutation frequency of EIF4G3 was the highest (3%), followed by LARP1 (2%), while nearly half of the regulators did not show any mutations. The summary of CNV showed that AGO2, NSUN2, METTL1, NCBP2 and NUDT3 were the top five regulators with highest CNV frequency, and amplification variations obviously higher than deletion (Figure 2B). The location of m^7^G regulators CNV on chromosomes was displayed in Figure 2C. To explore whether the genetic variation affect the expression level of m^7^G regulators in LUAD patients, the mRNA expression levels of these regulators were further analyzed between normal and LUAD samples, which indicated that most of these regulators were dysregulated in LUAD samples (Figure 2D). Moreover, univariate (Supplementary Figure S1A) and multivariate (Supplementary Figure S1B) Cox regression analyses further identified four independent poor prognostic factors (NUDT11, NUDT4, LARP1 and NCBP2) and two protective factors (EIF4E3, NUDT10) for LUAD patients. A complex regulatory network depicted the regulatory relationship (Supplementary Table S3) among m^7^G regulators and their prognostic significance for LUAD patients (Figure 2E). We found that most of regulators displayed a remarkably positive correlation in expression, whereas a few negative correlations among EIF4G3 and AGO2/NSUN2/WDR4/METTL1/LSM1, NUDT16 and LSM1/METTL1. In brief, the genetic alteration and expression level of m^7^G regulators are highly heterogeneous and significantly correlated with prognosis, indicating that the expressional alteration of m^7^G regulators played a crucial role in the LUAD occurrence and development.

### Identification of m^7^G methylation modification patterns mediated by 24 regulators

Based on the expression of 24 m^7^G regulators, three distinct m^7^G modification patterns were identified using unsupervised consensus clustering with optimal clustering stability, including 230 patients in pattern A, 262 patients in pattern B and 325 patients in pattern C (Supplementary Figures S2A–F). We named these patterns “m^7^Gcluster” A-C. Principal component analysis (PCA) confirmed the significant distinction existed on the m^7^G regulators expression among these m^7^G modification patterns (Supplementary Figures S2G). The expression patterns of m^7^G regulators and comparison of baseline clinicopathological characteristics in the three m^7^Gclusters were shown by the heatmap (Supplementary Figures S2H). m^7^Gcluster A exhibited high expression of METTL1, WDR4, NSUN2, DCPS, NUDT3, AGO2, EIF4E, LARP1, NCBP1, NCBP2, EIF4G3, LSM1; m^7^Gcluster B was characterized by decreased expression in almost regulators, except for METTL1, LSM1 and SNUPN; m^7^Gcluster C exhibited high expression of DCP2, NUDT16, CYFIP1, EIF4E3, and IFIT5.

### Biological behaviors and the tumor microenvironment characterization in distinct m^7^G modification patterns

**FIGURE 3:**
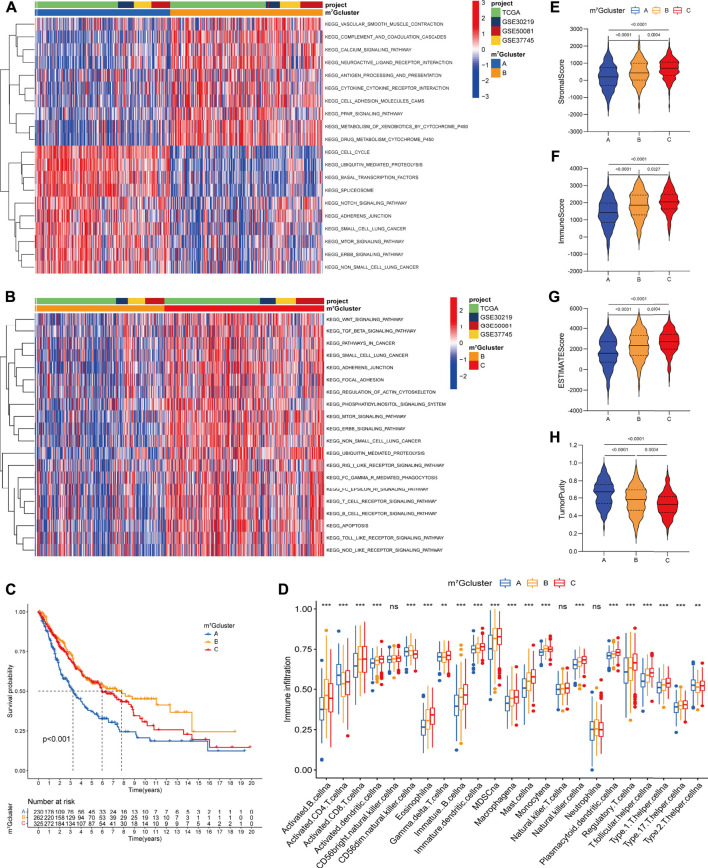
Biological characteristics and tumor microenvironment characterization of each m^7^G modification pattern. **(A,B)** The heatmap visualizes the enrichment of biological processes using GSVA analysis in distinct m^7^G modification patterns; **(A)** m^7^Gcluster A vs. m^7^Gcluster B; **(B)** m^7^Gcluster B vs. m^7^Gcluster C. Activated pathways are colored red and inhibited pathways are colored blue. The LUAD cohorts are used as sample annotations. **(C)** Survival analyses for the three m^7^G clusters based on 817 patients with LUAD from four cohorts (TCGA-LUAD, GSE30219, GSE50081, GSE37745). **(D)** The abundance of 23 TME infiltrating cells in the three m^7^G clusters. The horizontal line indicates the median, the lower and upper boundaries of the boxes the interquartile range, and the dots the outliers. Asterisks indicate statistical significance, *p < 0.05, **p < 0.01, ***p < 0.001. **(E–H)** Violin plots show differences in the **(E)** immune score, **(F)** stromal score, **(G)** ESTIMATE score and **(H)** tumor purity between distinct m^7^G clusters.

To identify the biological significance of distinct m^7^G modification patterns, we performed GSVA enrichment analysis (Supplementary Table S4). Specifically, m^7^Gcluster A was enriched in common oncogenic signaling pathways (e.g., mTOR, Notch and NSCLC signaling pathway), while lacked immune activation process (e.g., cytokine-cytokine receptor interaction, antigen processing and presentation), leading to the activation of abnormal biological characteristics including cell cycle, basal transcription factors, spliceosome, etc. (Figure 3A). On the contrary, in m^7^Gcluster B, various oncogenic signaling pathways such as mTOR, Notch and cancer associated pathways were strikingly suppressed, and immune activation process were significantly activated (Figures 3A,B). As expected, patients in m^7^Gcluster A showed the worst prognosis, whereas patients in m^7^Gcluster B had the best prognosis (Figure 3C). Significantly, m^7^Gcluster C was significantly enriched in innate immune activation process (such as Fc epsilon RI, Nod like receptor, Toll like receptor, and Fc gamma R-mediated phagocytosis signaling pathways) (Figure 3B), while patients with this m^7^G modification pattern did not show a matching survival advantage. We speculated that this result may be related to high-level enrichment of stromal interactions pathways (such as ERBB, Wnt, TGF beta, Adherens Junction, Focal Adhesion and Regulation of Actin Cytoskeleton pathways), as well as B cell receptor (BCR) and T cell receptor (TCR) which could upregulate B and T cells activation threshold.

To further explore the TME characterization, we compare the difference in immune cell infiltration among distinct m^7^G modification patterns (Supplementary Table S5; Figure 3D). Increased adaptive (e.g., B cell, CD8^+^ T cell, T helper cell) and innate (e.g., macrophage, NK cell, dendritic cell) immune cell infiltration was exhibited in m^7^Gcluster B and C as compared to m^7^Gcluster A. To our surprise, m^7^Gcluster C was remarkably rich in innate immune cell infiltration including dendritic cell, eosinophil, gamma delta T cell, macrophage, mast cell, natural killer cell, and cell types associated with immune suppression such as MDSC and regulatory T cell. Additionally, the ESTIMATE algorithm revealed the lowest level of stromal and immune cell infiltration in m^7^Gcluster A, while revealed the highest level in m^7^Gcluster C (Figures 3E–H). Based on the above results and previous research (Chen and Mellman, 2017), We could summarize that the three m^7^G modification patterns corresponded to three different immunophenotypes. Strikingly, m^7^Gcluster A was identified as immune-desert phenotype, characterized by suppressed immune-related pathways and deficient immune cell infiltration. m^7^Gcluster B was identified as immune-inflamed phenotype, characterized by immune activation and high level of adaptive immune cells infiltration. More importantly, we found that the TME characterization of m^7^Gcluster C was consistent with the immune-excluded phenotype described by Chen (Chen and Mellman, 2017), which was characterized by enhanced tumor stroma activity and abundant innate immune cells trapped in surrounding tumor cell nests.

### Identification of m^7^G modification-related genomic subtypes and transcriptome characterization

**FIGURE 4:**
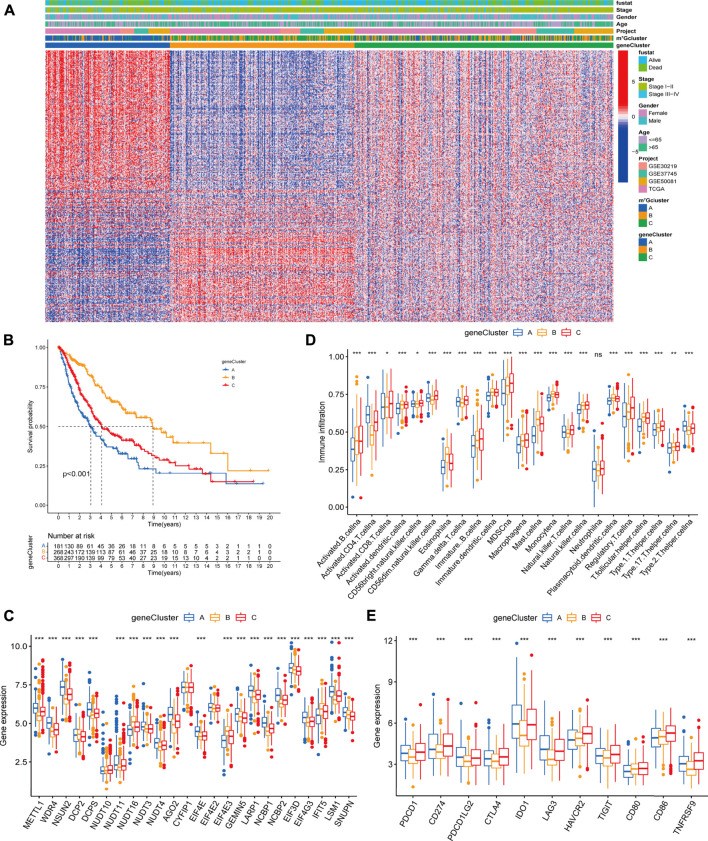
Clinicopathologic characteristics, genomic profiling and tumor microenvironment characteristics among distinct m^7^G modification-related genomic subtypes. **(A)** The heatmap visualizes the gene expression levels across the whole genome and comparison of baseline clinicopathological characteristics in each sample. Blue represents low expression and red represent high expression. **(B)** Survival analyses for the three gene clusters based on 817 patients with LUAD from four cohorts (TCGA-LUAD, GSE30219, GSE50081, GSE37745). **(C)** Difference in the m^7^G regulators expression among three gene clusters. **(D)** Difference in the abundance of 23 TME infiltrating cells among three m^7^G gene clusters. **(E)** Difference in the immune-checkpoint related gene expression among three gene clusters. The horizontal line indicates the median, the lower and upper boundaries of the boxes the interquartile range, and the dots the outliers. Asterisks indicate statistical significance, *p < 0.05, **p < 0.01, ***p < 0.001.

In order to further explore the potential biological significance of distinct m^7^G modification patterns, we distinguished 2071 m^7^G-related differentially expressed genes (DEGs) (Supplementary Figure S3A, Supplementary Table S6). Subsequently GO enrichment analysis of m^7^G-related DEGs revealed significant enrichment of biological processes related to m^7^G modification and immune system (Supplementary Table S7, Supplementary Figure S3B), which confirmed that m^7^G modification played a critical role in immune modulation of the TME. To clarify the potential mechanisms, we further performed unsupervised consensus clustering based on the 401 prognosis m^7^G-related DEGs (Supplementary Table S8) and classify the entire LUAD cohort into three main m^7^G-related genomic subtypes (named as m^7^G gene cluster A-C), including 181 patients in cluster A, 268 patients in cluster B and 368 patients in cluster C (Supplementary Figures S4A–F). Signature genes expression level and baseline clinicopathological characteristics for the different clusters were displayed in Figure 4A. We found that m^7^G gene cluster A and B showed opposite gene expression patterns, and patients with alive status or clinical stage I-II were mainly concentrated in the cluster B. Kaplan-Meier analysis indicated that gene cluster A exhibited poorer prognosis, while gene cluster B exhibited better prognosis, and gene cluster C exhibited intermediate prognosis (Figure 4B). Moreover, prominent differences were observed in the expression of m^7^G regulators among the three gene clusters (Figure 4C), which demonstrated again that m^7^G ​​modification modulate the genomic phenotype of LUAD patients.

To explore the relationship between m^7^G-related genomic features and the tumor immune microenvironment, we examined the immune cell infiltrating characteristics and immune-checkpoint related gene (ICG) expression in three gene clusters. As shown in Figures 4D,E, gene cluster A was characterized by low levels of immune cell infiltration and upregulated ICG expression. Conversely, gene cluster B was characterized by high levels of immune cell infiltration and downregulated ICB expression. Remarkably, gene cluster C exhibited high immune cell infiltration level, while high immune checkpoint related mRNAs expression, which might be relevant to the poor prognosis.

### Construction and evaluation of m^7^Gscore, one m^7^G modification quantification system

**FIGURE 5:**
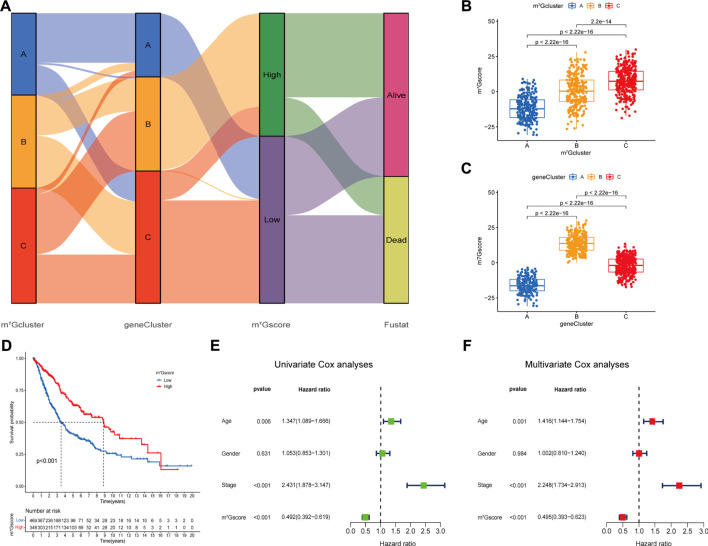
Construction of the m^7^G signature and correlation of m^7^Gscore with clinicopathological features. **(A)** Alluvial diagram shows the changes of m^7^G clusters, gene clusters, m^7^Gscore and survival state. **(B)** Differences in m^7^Gscore among three m^7^G clusters (*p* < 0.001, Kruskal-Wallis test). **(C)** Differences in m6Ascore among three gene clusters (*p* < 0.001, Kruskal-Wallis test). **(D)** Survival analyses for low (469 cases) and high (348 cases) m^7^Gscore groups in the four cohorts using Kaplan-Meier curves (*p* < 0.0001, Log-rank test). **(E,F)** Univariate **(E)** and multivariate **(F)** analyses for m^7^Gscore using the Cox regression model.

Regrettably, the above conclusions were generated based on group-level analyses, the characteristics of m^7^G modification in individual patients were limited. Considering the heterogeneity of m^7^G modification, we constructed a scoring system (named as m^7^Gscore) based on prognosis m^7^G-related DEGs to accurately quantify and predict the individual tumors m^7^G modification pattern (Supplementary Table S9). Patients were classified into high m^7^Gscore group (*n* = 348) and low m^7^Gscore group (*n* = 469) using the optimal cut-off value 2.984 identified by the “surv_cutpoint” function from the “survminer” R package. An alluvial diagram was generated to depict the distribution transitions of individual patients among the m^7^Gclusters, m^7^G gene clusters and m^7^Gscore groups (Figure 5A). Nonparametric Kruskal-Wallis (K-W) test was performed to reveal the difference in m^7^Gscore among distinct m^7^Gclusters and m^7^G gene clusters. We noticed that m^7^Gcluster A presented the lowest median score, whereas m^7^Gcluster C presented the highest (Figure 5B), which suggested that low m^7^Gscore might be closely associated with deficient immune cell infiltration while high m^7^Gscore might be linked to stromal activation. Additionally, m^7^G gene cluster B had significantly higher m^7^Gscore than the other two clusters, while m^7^G gene cluster A showed the lowest median score (Figure 5C). Moreover, K-M analysis indicated that high m^7^Gscore conferred a significant survival benefit (*p* < 0.001, Figure 5D). Subsequent subgroup analyses (Supplementary Figures S5E–J) showed that the prognostic value of m^7^Gscore remained statistically significant for each subgroup based on gender (male, female), age (≤65, >65), and clinical stage (I-II, III-IV). m^7^Gscore also showed the prognostic value in different datasets (Supplementary Figures S5A–D). Additionally, As shown in Supplementary Figures S5H–L, patients with high m^7^Gscore exhibited a significantly higher percentage of alive status (68%), and patients who died had remarkably lower m^7^Gscore (*p* < 0.001). Univariate (Figure 5E) and multivariate (Figure 5F) Cox analyses confirmed m^7^Gscore as a robust and independent prognostic biomarker for evaluating patient outcomes [HR: 0.495 (0.393–0.623), *p* < 0.001].

**FIGURE 6:**
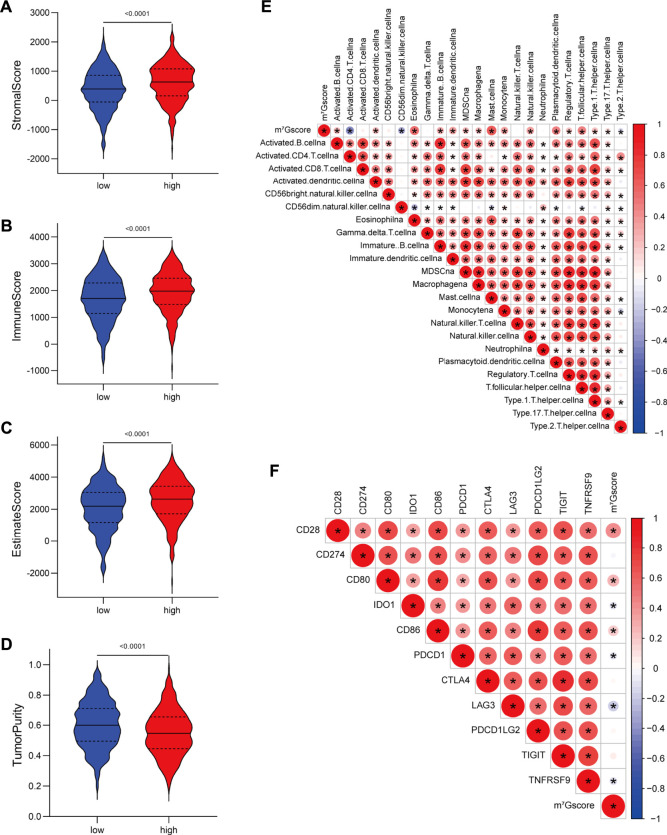
Correlations between m^7^G signature and the tumor microenvironment characteristics. **(A–D)** Violin plots show differences in the **(A)** immune score, **(B)** stromal score, **(C)** estimate score and **(D)** tumor purity between low and high m^7^Gscore groups. **(E)** Correlations between m^7^Gscore and the abundance of 23 TME infiltrating cells. **(F)** Correlations between m^7^Gscore and immune-checkpoint related gene expression. Positive correlation was marked with red and negative correlation with blue. The circle color represents Spearman coefficient value, the size of circle is inversely proportional to the *p*-value, and the asterisk stands for *p* < 0.05.

Furthermore, we assessed the relationship between m^7^Gscore and the TME cell infiltration to explore whether m^7^G modification quantification system can reflect the TME heterogeneity. The ESTIMATE algorithm indicated that high m^7^Gscores were significantly associated with enhanced levels of immune and stromal cell infiltration as well as low tumor purity (*p* < 0.001; Figures 6A–D). Correlation analysis of immune cell infiltration and m^7^Gscore (Figure 6E) indicated that m^7^Gscore was significantly positively correlated with most types of innate immune cells (e.g., eosinophil, dendritic cell, mast cell) and B cell, whereas negatively correlated with active CD4 T cell and CD56dim NK cell. In addition, m^7^Gscore was negatively correlated with immunosuppression-related ICGs (including IDO1, PDCD1, LAG3, TNFRSF9), whereas positively correlated with CD28^−^CD80/86 (Figure 6F) which provides co-stimulatory signals for T-cell activation (Esensten et al., 2016; Ma et al., 2021a). Considering the above results, high m^7^Gscore indicated increased immune and stromal cell infiltration, while low m^7^Gscore was correlated with decreased immune cell infiltration and great immunosuppression. m^7^Gscore could reflect the m^7^G modification pattern of individual LUAD patients to further evaluate the TME characterization.

### Analysis of tumor somatic mutation in patients with different m^7^Gscores

**FIGURE 7:**
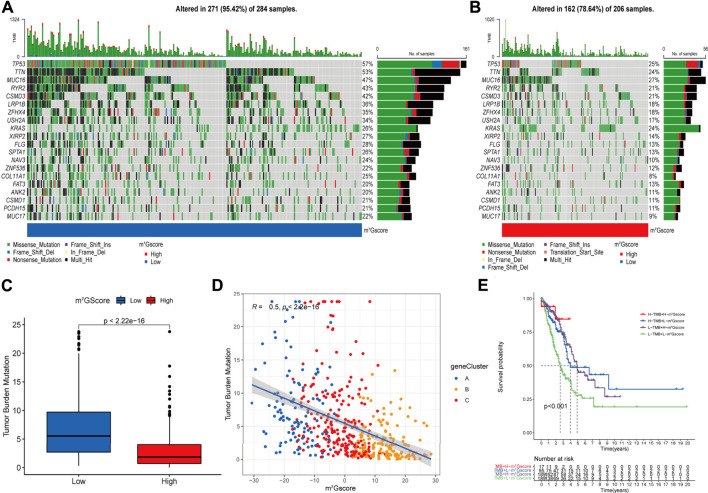
The correlation of m^7^Gscore and the tumor somatic mutation. **(A,B)** The waterfall plot of tumor somatic mutation in LUAD patients with low m^7^Gscore **(A)** and high m^7^Gscore **(B)**, each column represents individual patients. The top barplot depicts tumor mutation burden (TMB) and mutation frequency in each gene is given on the right. The right barplot depicts the proportion of each variant type. The stacked barplot below depicts fraction of conversions in each sample. **(C,D)** The relationship between the m^7^Gscore and TMB. **(E)** The Kaplan–Meier curves of the OS of subgroup patients stratified by the m^7^Gscore and TMB.

Accumulated evidence demonstrated that tumor mutation burden (TMB) is an emerging biomarker of response to immune checkpoint blockade (ICB) therapy (Yarchoan et al., 2017; Ready et al., 2019), we analyzed the landscape of tumor somatic mutation among patients with different m^7^Gscore to indirectly reflect the immunotherapeutic outcomes in TCGA-LUAD cohort. The waterfall plots suggested that low m^7^Gscore group exhibited more extensive tumor somatic mutation than the high m^7^Gscore group, with the rate of the 20th most significant mutated gene 22% versus 9% (Figures 7A,B). As shown in Figures 7C,D, the m^7^Gscore exhibited a significant negative correlation to TMB. Subsequent Kaplan-Meier survival analysis indicated that patients with a high TMB level had better OS than those with a low TMB level in low m^7^Gscore group (Figure 7E). The above results demonstrated that m^7^Gscore could effectively reflect the TMB level of LUAD, which indirectly indicated the values of distinct m^7^G modification patterns in predicting ICB therapy outcomes.

### Prediction of immunotherapy and chemotherapy response

**FIGURE 8:**
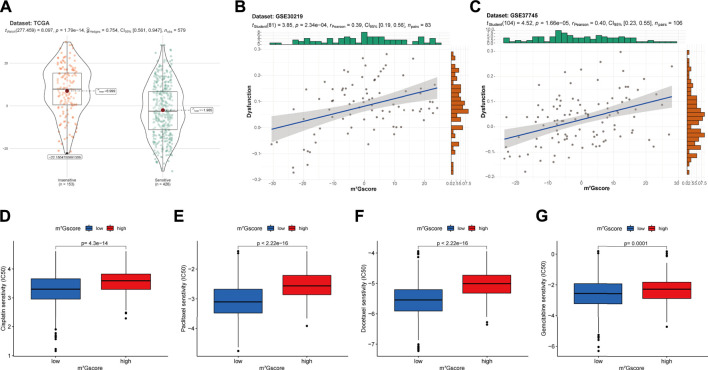
The correlation of m^7^Gscore and the therapeutic response. **(A)** The difference in m^7^Gscore between sensitive and insensitive groups divided by TIDE algorithm in TCGA-LUAD cohort. **(B,C)** The correlation of m^7^Gscore and the T cell dysfunction signature in GSE30219 **(B)** and GSE37745 **(C)** cohorts. **(D–G)** Differences in the IC50 of cisplatin **(D)**, paclitaxel **(E)**, docetaxel **(F)**, gemcitabine **(G)** between low and high m^7^Gscore groups. IC50, 50% inhibitory concentration, which negatively correlated with drug responsiveness.

Based on the TIDE (Tumor Immune Dysfunction and Exclusion) algorithm, patients in TCGA-LUAD cohort were classified into insensitive and sensitive groups. As shown in Figure 8A, m^7^Gscores were significantly higher in the insensitive group than in the sensitive group (*p* < 0.001). Similarly, m^7^Gscores were significantly positively correlated with T cell dysfunction scores in GSE30219 (R = 0.39, *p* < 0.001; Figure 8B) and GSE37745 (R = 0.40, *p* < 0.001; Figure 8C) cohorts, which suggested that patients with higher m^7^Gscores had poor immunotherapy response rates. Moreover, there were marked increases in the IC50 to cisplatin (Figure 8D, *p* < 0.001), paclitaxel (Figure 8E, *p* < 0.001), docetaxel (Figure 8F, *p* < 0.001) and gemcitabine (Figure 8G, *p* < 0.01) in high m^7^Gscore group, which indicated the poor efficacy to these drugs in patients with high m^7^Gscores compared to patients with low m^7^Gscores. Together, m^7^Gscore could effectively predict the response to chemotherapy and immunotherapy for LUAD patients.

## Discussion

With the rapid advancement of deep sequencing and large-scale profiling (Enroth et al., 2019; El Allali et al., 2021), accumulating evidence has demonstrated that m^7^G modification is critical for maintaining the physiological conditions of cells and organisms (Pei and Shuman, 2002; Lindstrom et al., 2003; Haag et al., 2015), while its aberrant distribution is closely related to tumor development and progression (Ma et al., 2021b). Moreover, recent studies have also confirmed that m^7^G may affect the distribution and function of immune cells (Zhang et al., 2021; Gao et al., 2022), such as T cells (Galloway et al., 2021). As most studies have focused on single regulator or single TME cell type, a comprehensive recognition of TME infiltration characterizations mediated by multiple m^7^G regulators is still lacking. Exploring the role of different m^7^G modification patterns in the TME cell infiltration will help to enhance our understanding of the TME antitumor immune response and guide novel immunotherapy strategies.

In this study, three m^7^G methylation modification patterns were identified based on 24 potential m^7^G regulators, which had significantly distinct TME cell infiltration characterizations. The m^7^G cluster A was characterized by suppressed immune-related functions and deficient immune cells infiltration, consistent with immune-desert phenotype; cluster B was characterized by immune activation and high level of adaptive immune cells infiltration, consistent with immune-inflamed phenotype; cluster C was characterized by enhanced tumor stroma activity and abundant innate immune cells, consistent with the immune-excluded phenotype. As mentioned in previous literatures (Gajewski et al., 2013; Joyce and Fearon, 2015; Chen and Mellman, 2017), the immune-inflamed tumors can demonstrate infiltration of large number of immune cells, especially T, B and monocytic cells, in the tumor parenchyma; the immune-excluded tumors are also characterized by the presence of abundant immune cells, but the immune cells are retained in the stroma surrounding nests of tumor cells rather than penetrate the parenchyma; the immune-desert tumors are associated with the immunological ignorance and paucity of immune cells in either the tumor parenchyma or the stroma. Significant enrichment of stromal interactions pathways in m^7^G cluster C and the characteristics of TME cell infiltration in each cluster corroborate the accuracy of our immunophenotypic classifications for distinct m^7^G modification patterns. Not surprisingly, m^7^G cluster C exhibits activated innate immunity but no matching survival advantage.

Furthermore, differentially expressed genes among the three modification patterns (named as m^7^G-related DEGs) were identified and demonstrated to be significantly associated with immune-related biological pathways. Three m^7^G-related genomic subtypes were identified based on 401 prognostic m^7^G-related DEGs, which were also significantly related to immune cell infiltration and activation. This further demonstrated the crucial role of m^7^G modification in modulating the TME landscape. Given the individual heterogeneity of m^7^G modification, it was critical to quantify m^7^G modification patterns in individual tumors. Therefore, we developed a novel scoring system (m^7^Gscore) to assess the m^7^G modification pattern of individual LUAD patients. The patients with immune-excluded and immune-inflamed tumor presented a higher m^7^Gscore, while the patients with immune-desert tumor presented a lower m^7^Gscore. Also, high m^7^Gscores were significantly associated with enhanced levels of immune and stromal cells infiltration. These results suggested m^7^Gscore was a reliable tool for assessing individual tumor m^7^G modification patterns, which could further indicate the immune phenotype in tumor environment. Additionally, m^7^Gscore was proved to be an independent prognostic factor, with lower m^7^Gscores indicating poorer prognosis.

Our results demonstrated the significantly negative correlations of m^7^Gscore with the expression of IDO1, PDCD1, and LAG3, which have been considered as important targets for cancer immunotherapy (Cyriac and Gandhi, 2018; Ruffo et al., 2019; Tang et al., 2021). There was also a markedly negative correlation between m^7^Gscore and tumor mutation burden (TMB). Growing evidence demonstrated that patients with high TMB had a greater clinical response to anti-PD-1/PD-L1 immunotherapy (Yarchoan et al., 2017; Ready et al., 2019). The Tumor Immune Dysfunction and Exclusion algorithm further showed that higher m^7^Gscore was associated with T cells dysfunction and exclusion, which directly reflect the efficacy to T cell-based immunotherapy (Jiang et al., 2018). Thus, the above results fully demonstrated that individual m^7^G modification pattern could be an effective indicator that estimate the responsiveness to Immune checkpoint blockade (ICB) therapy. Many reports have revealed the interaction between chemotherapy and immunotherapy, and the differences in immune and stromal cell infiltration in TME jointly affect resistance to chemotherapy (Wang et al., 2016; Zhu et al., 2021). Fu et al. (2018) have reported the immunotypes could predict the efficacy of patients to adjuvant chemotherapy. In this study, patients with higher m^7^Gscore exhibited poor efficacy to several first-line chemotherapy drugs (including cisplatin, paclitaxel, docetaxel and gemcitabine) for LUAD, and this might due to lower T cell infiltration and higher stromal cell infiltration.

In a word, m^7^Gscore can act as an effective tool to evaluate the individual m^7^G modification pattern and the corresponding immune phenotypes for LUAD patients, further to guide treatment decisions in clinical practice. m^7^Gscore can also be a potential prognostic biomarker for predicting survival. More importantly, m^7^Gscore may guide the clinicians in predicting the clinical response to ICB therapy and the efficacy of adjuvant chemotherapy. Modifying the m^7^G modification patterns by targeting m^7^G regulators or m^7^G-related genes may improve unfavorable TME cell infiltrating characterization, that may contribute to the development of novel immunotherapy target or optimization of combination therapy strategies. These findings offered new insights for identifying distinct immune phenotypes and developing individualized cancer immunotherapy.

Despite the important strengths of this study, several limitations should be noted. First, due to the limited clinicopathological parameters in public datasets, there would be potential bias when the m^7^Gscore acted as a prognosis biomarker. Second, we did not evaluate the location of immune and stromal cell infiltration in the TME. Thirdly, we could not directly analysis the correlation between m^7^Gscore and LUAD patients’ response to therapy due to the lack of treatment-related information. Finally, our findings were carried out based on a bioinformatics analysis, and further experimental validation is warranted.

## Conclusion

In conclusion, this study revealed the non-negligible role of m^7^G modification in TME heterogeneity and complexity. Identifying m^7^G modification patterns helps predict clinical response to ICB therapy and efficacy of adjuvant chemotherapy. We believe that the assessment of individual tumor m^7^G modification pattern will contribute to a more comprehensive understanding of TME characterization and facilitate the development of novel immunotherapy strategies.

## Data Availability

The original contributions presented in the study are included in the article/Supplementary Material, further inquiries can be directed to the corresponding authors.
