# Supplementary figures and images for: Comprehensive analysis of m7G modification patterns based on potential m7G regulators and tumor microenvironment infiltration characterization in lung adenocarcinoma
Source: Front Genet. 2022 Sep 29;13:996950. doi: 10.3389/fgene.2022.996950 (PMC9559715; doi:10.3389/fgene.2022.996950)

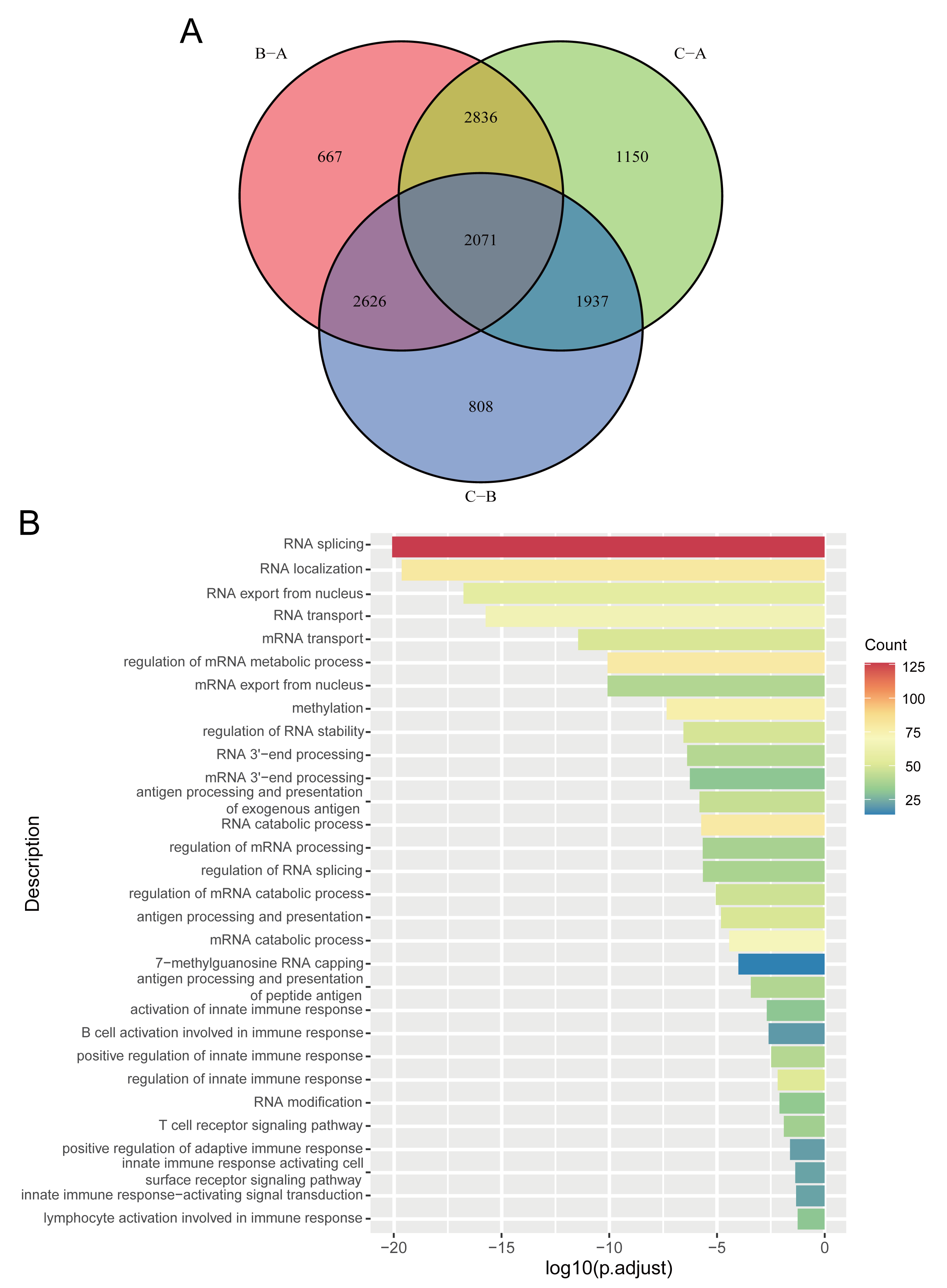

Supplement: Supplementary file 1 [file Image3.TIF]

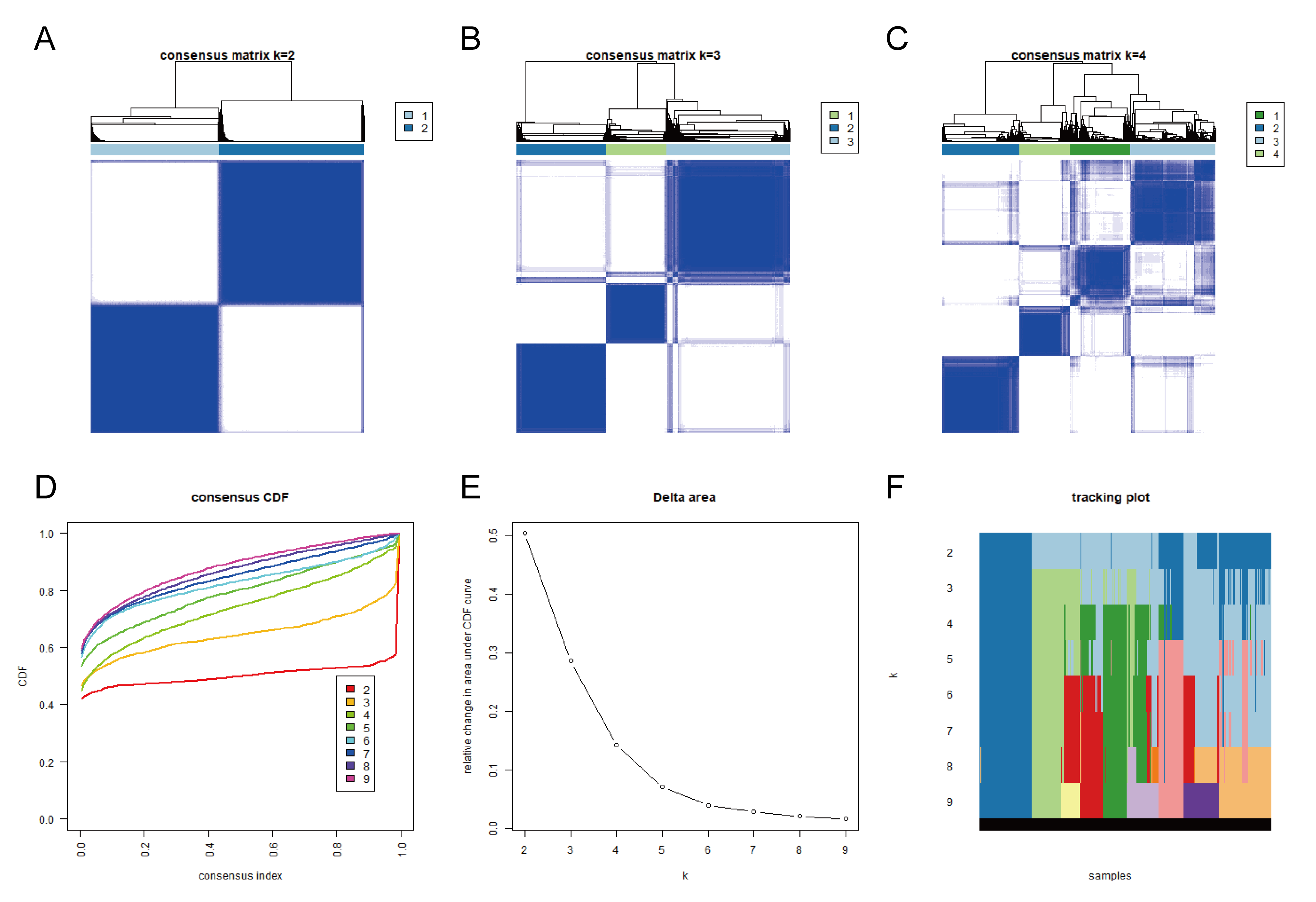

Supplement: Supplementary file 2 [file Image4.TIF]

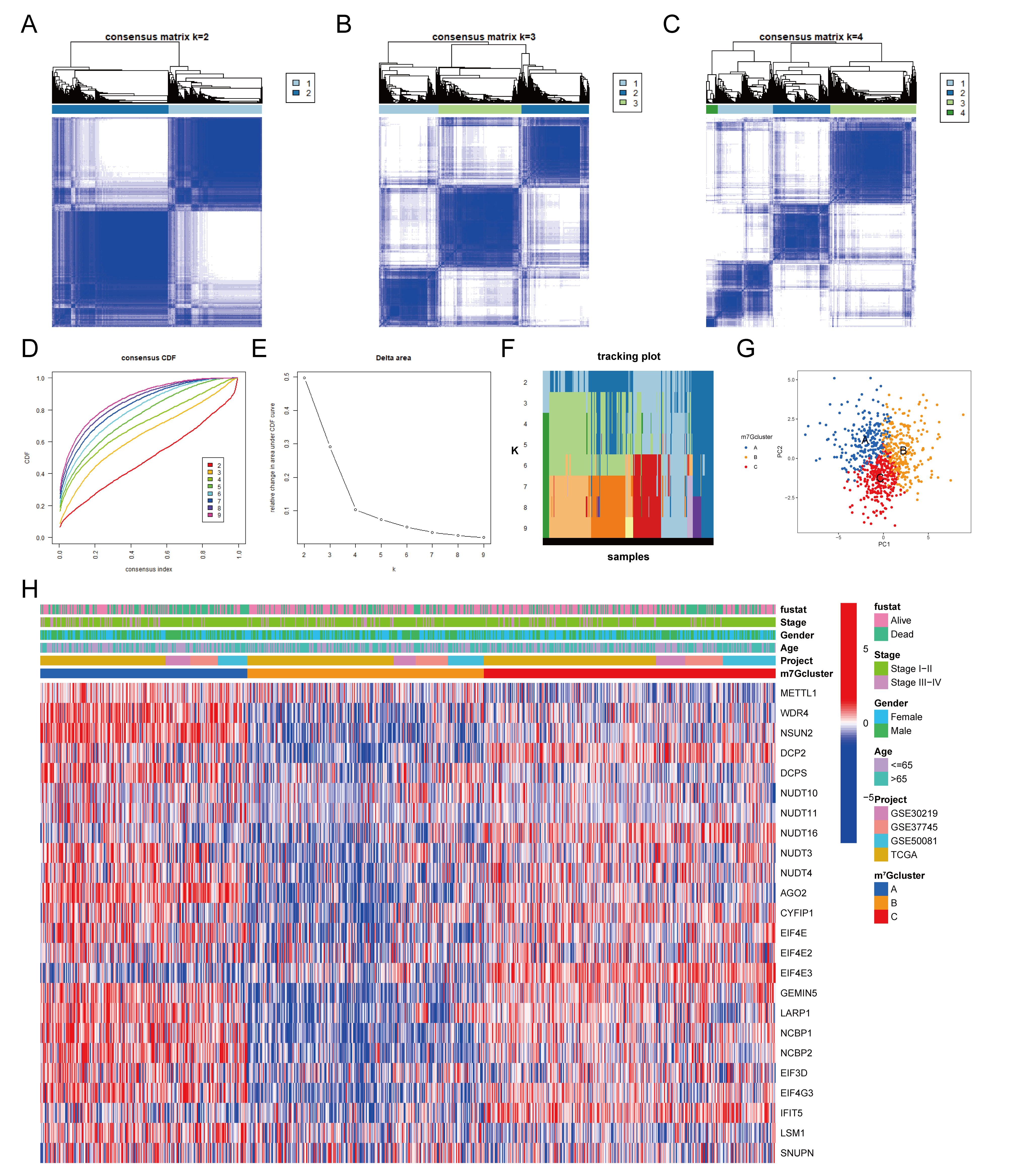

Supplement: Supplementary file 3 [file Image2.TIF]

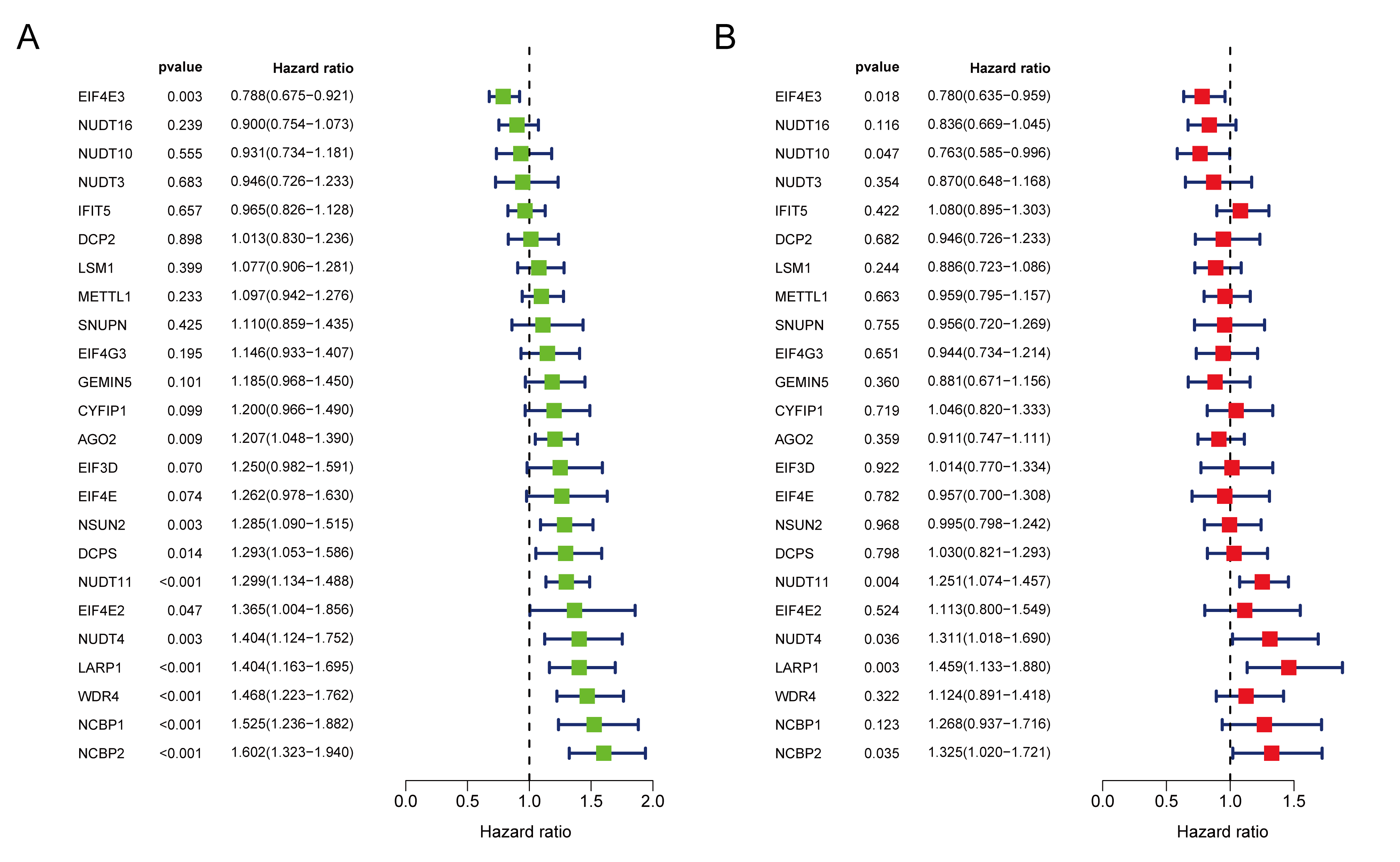

Supplement: Supplementary file 4 [file Image1.TIF]

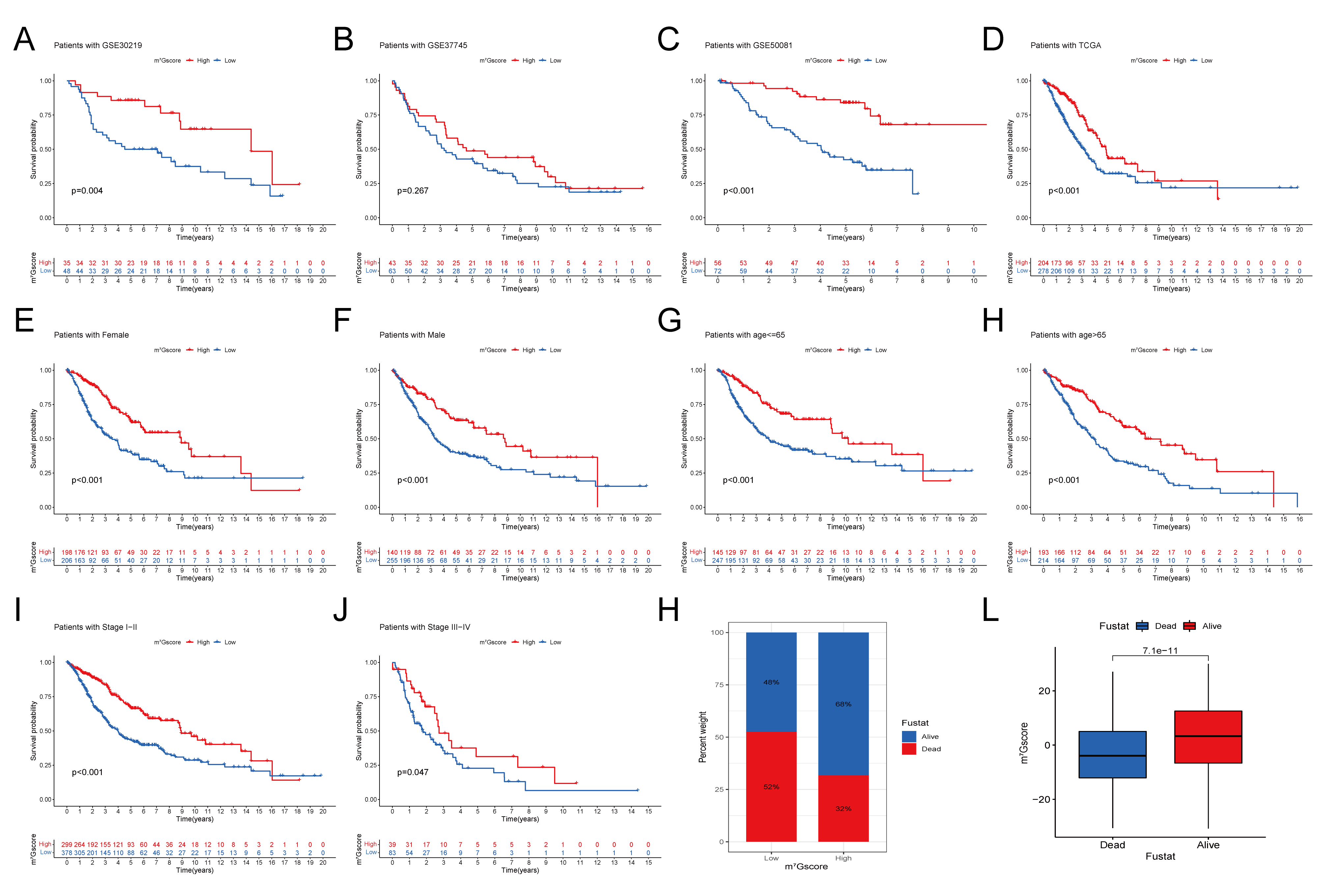

Supplement: Supplementary file 6 [file Image5.TIF]
