# Supplementary material for: Comprehensive analysis of m7G modification patterns based on potential m7G regulators and tumor microenvironment infiltration characterization in lung adenocarcinoma
Source: Front Genet. 2022 Sep 29;13:996950. doi: 10.3389/fgene.2022.996950 (PMC9559715; doi:10.3389/fgene.2022.996950)
Supplement: Supplementary file 7 [file DataSheet1.docx]

**Supplementary Figure 1.** **The prognostic analysis of m7G regulators.**
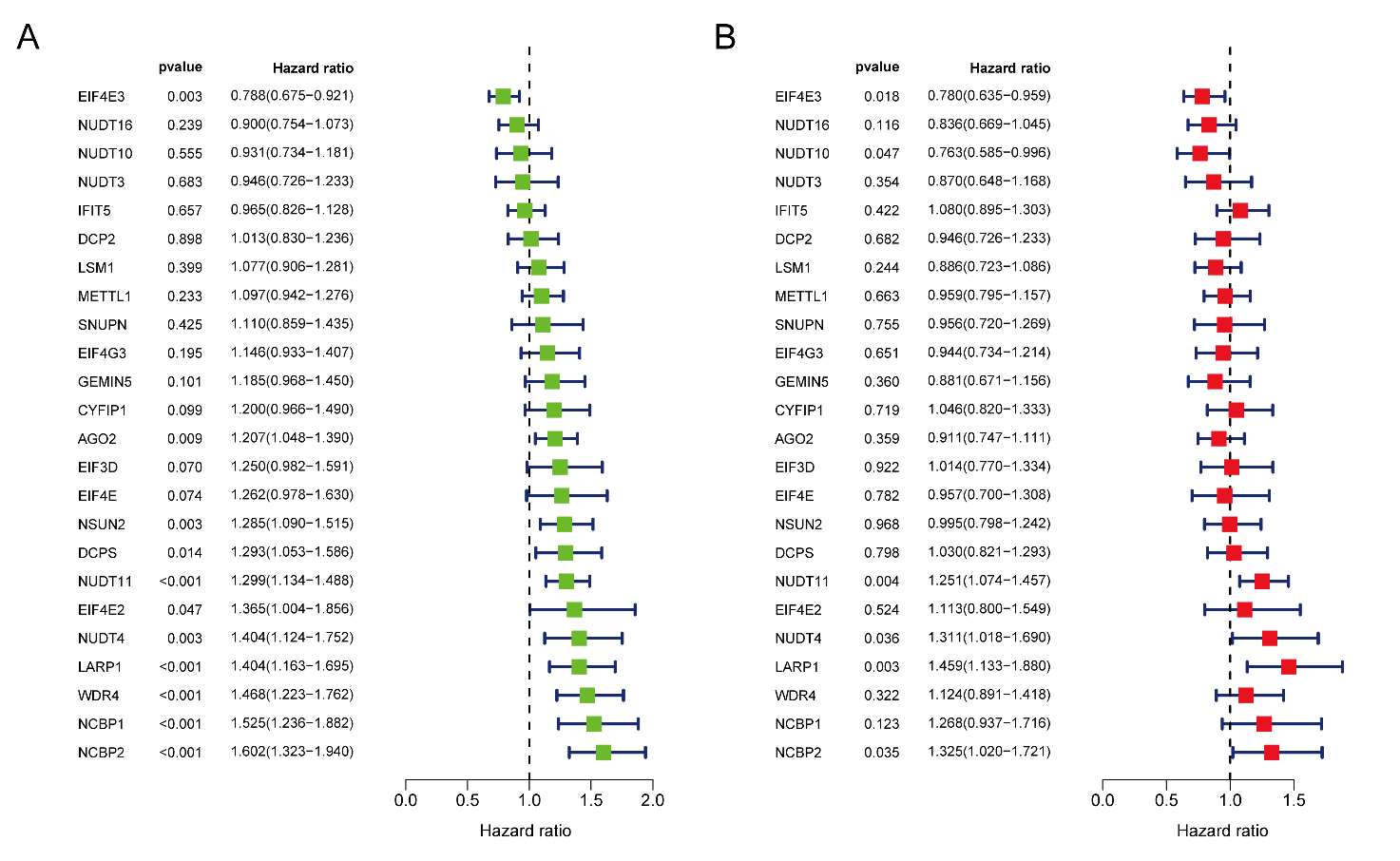


A-B: Univariate (A) and multivariate (B) analyses for 24 m7G regulators in the four LUAD cohorts using the Cox regression model. A hazard ratio of <1 indicates a favorable influence on survival and a hazard ratio of >1 indicates an unfavorable influence on survival.

**Supplementary Figure 2.**  **Unsupervised consensus clustering identified three clusters with distinct m7G modification patterns.**
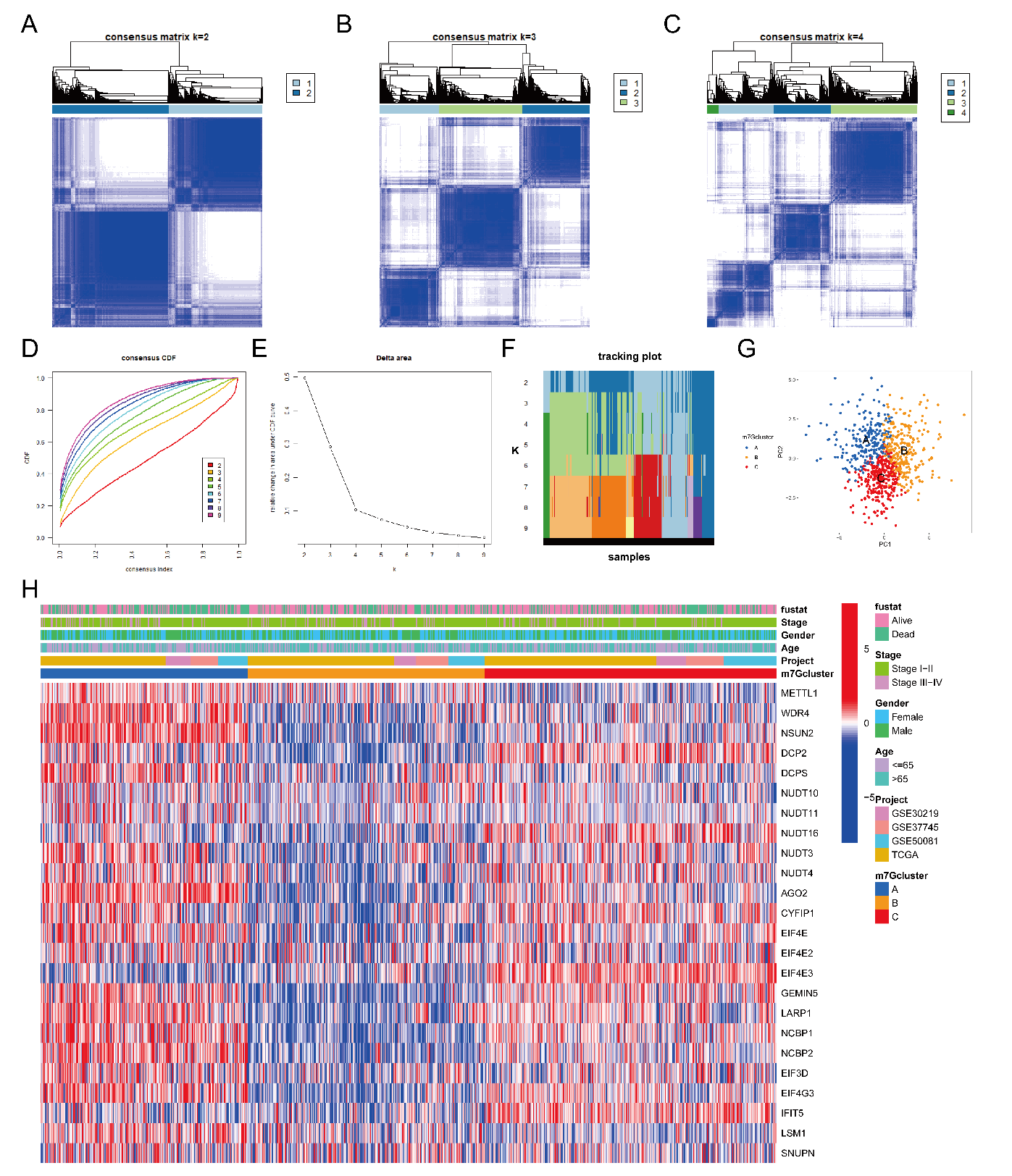


(A-C) Consensus matrices of the four LUAD cohorts for k = 2 – 4. (D) Consensus clustering cumulative distribution function (CDF) for k=2 – 9; (E) relative change in area under CDF curve for k=2 – 9; (F) distribution of each sample for k=2 – 9; (G) Principal component analysis for the transcriptome profiles of the three m7G clusters. (H) The heatmap visualizes the expression levels of m7G regulators and comparison of baseline clinicopathological characteristics in each sample. Blue represents low expression and red represent high expression.

**Supplementary Figure 3. Identification and functional annotation of m7G-related differentially expressed genes (DEGs).**


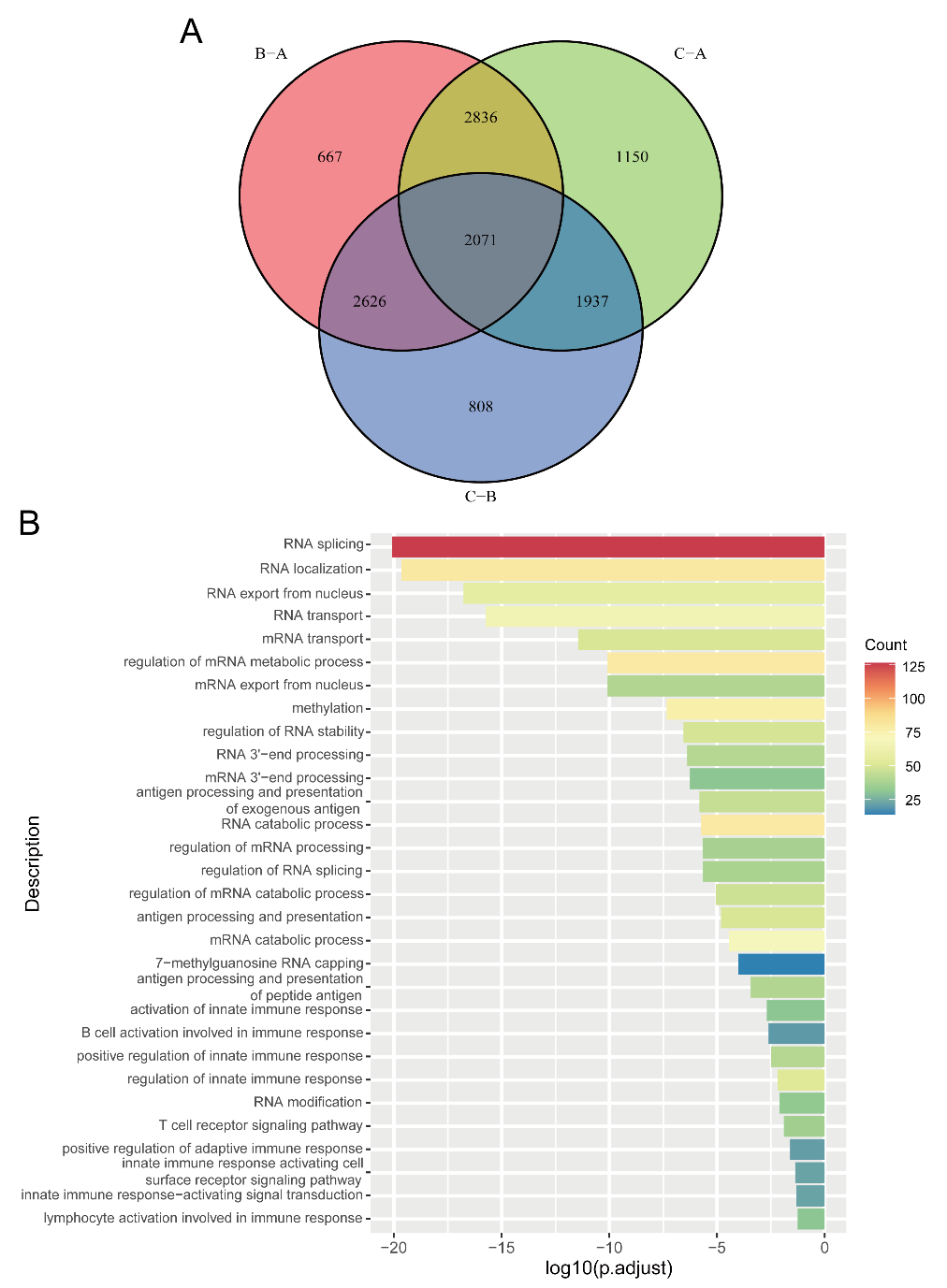


(A) 2071 m7G-related DEGs were shown in the venn diagram. (B) Functional annotation for m7G-related DEGs using GO enrichment analysis. The color depth of the barplots represented the number of genes enriched.

**Supplementary Figure 4. Unsupervised consensus clustering identified three clusters with distinct m7G modification-related genomic subtypes.**


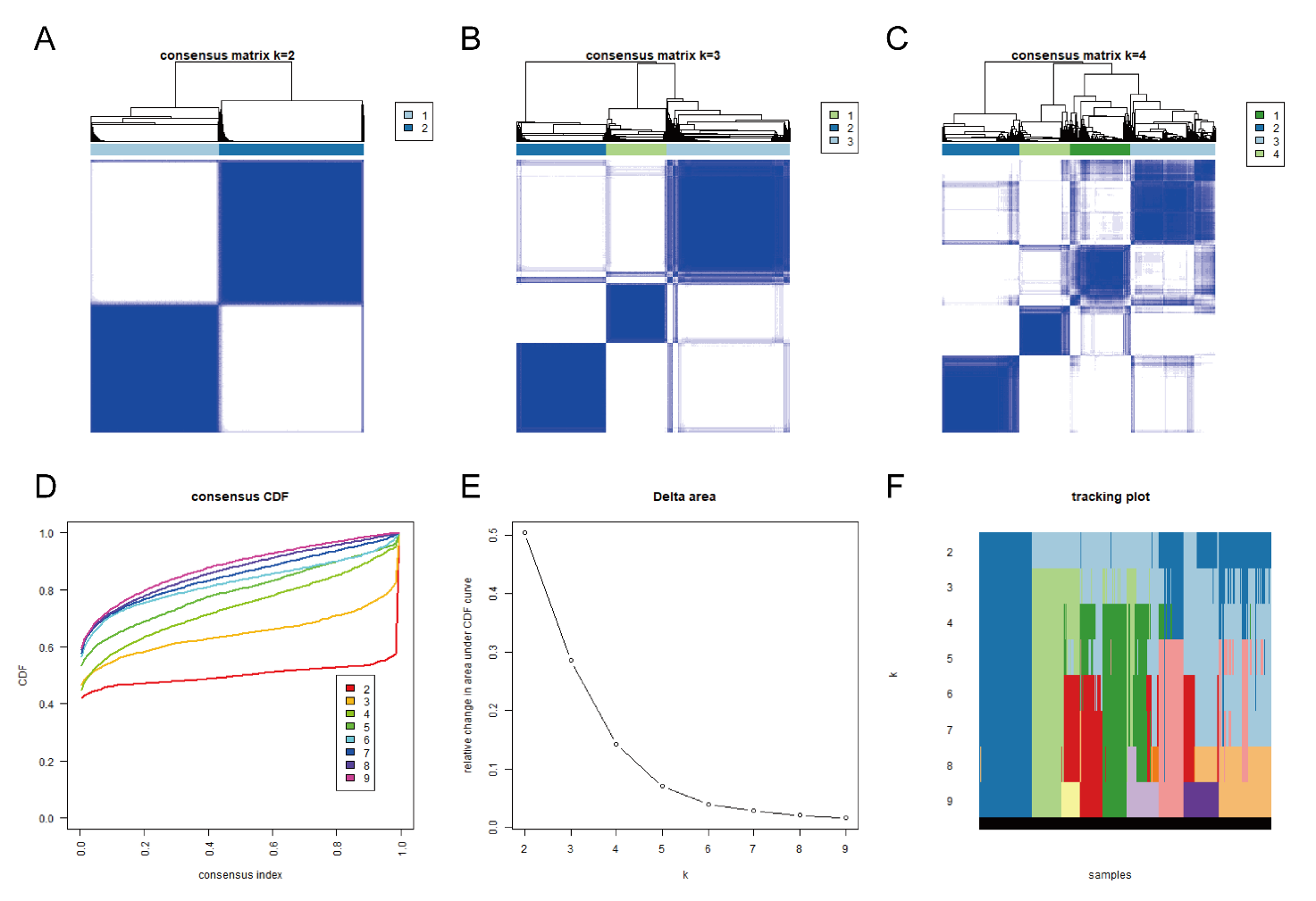


(A-C) Consensus matrices of the four LUAD cohorts for k = 2 – 4. (D) Consensus clustering cumulative distribution function (CDF) for k=2 – 9; (E) relative change in area under CDF curve for k=2 – 9; (F) distribution of each sample for k=2 – 9.

**Supplementary Figure 5. Prognostic value of m7Gscore in different subgroups.**


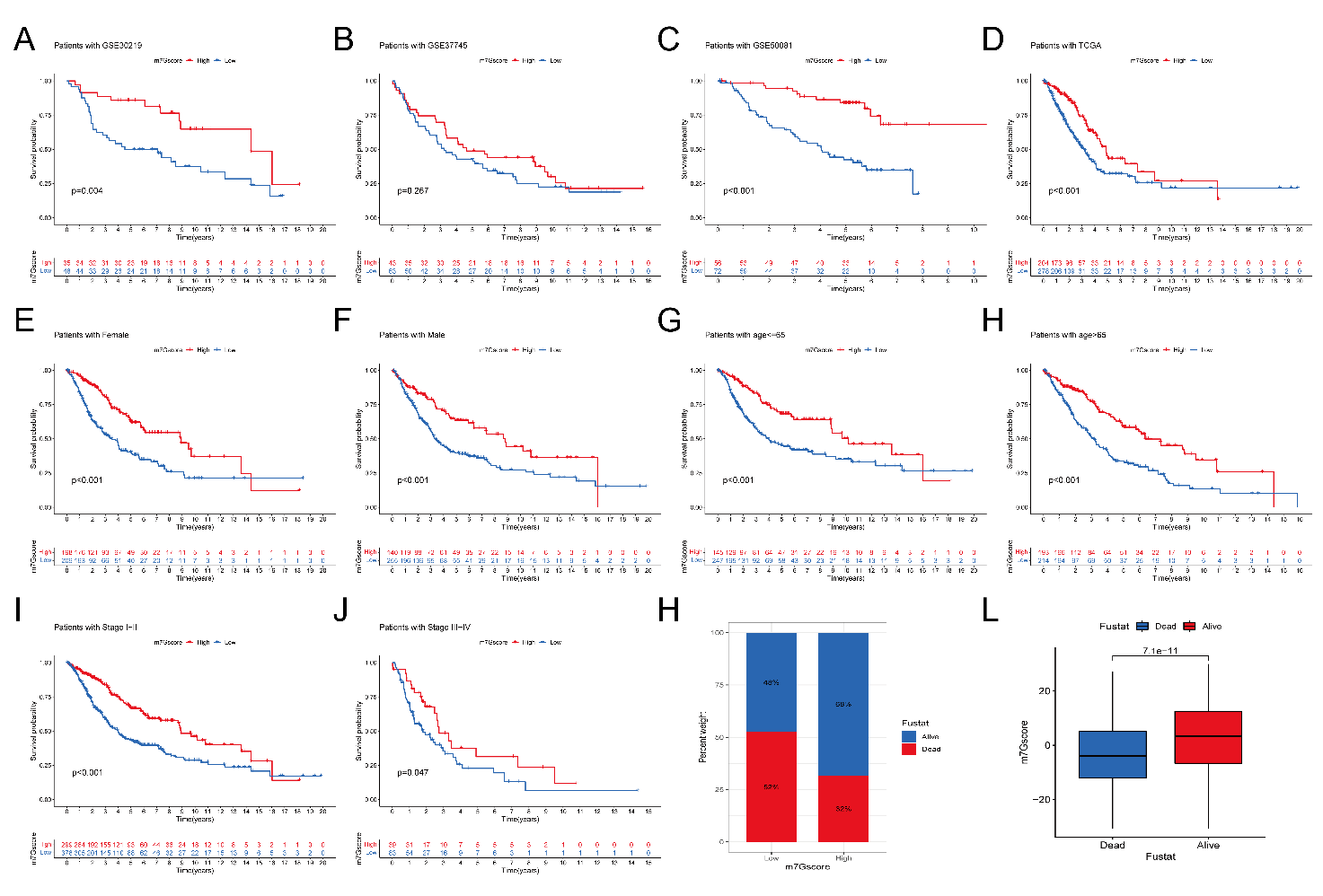


(A-D) Survival analysis of m7Gscore in distinct LUAD cohorts: (A) GSE30219; (B) GSE37745; (C) GSE50081; (D) TCGA-LUAD. (E-J) Survival analysis of m7Gscore in distinct subgroups with different clinical characteristics: (E) female; (F) male; (G) age≤ 65; (H) age> 65; (I) Stage I-II; (J) Stage III-IV. (H) The proportion of deaths in low or high m7Gscore groups. (L) Differences in m7Gscore between death and survival, the lines in the boxes represented median value (Wilcoxon test).
